# Semantically linking molecular entities in literature through entity relationships

**DOI:** 10.1186/1471-2105-13-S11-S6

**Published:** 2012-06-26

**Authors:** Sofie Van Landeghem, Jari Björne, Thomas Abeel, Bernard De Baets, Tapio Salakoski, Yves Van de Peer

**Affiliations:** 1 Department of Plant Systems Biology, Flanders Institute for Biotechnology (VIB), B-9052 Gent, Belgium; 2 Department of Biotechnology and Bioinformatics, Ghent University, B-9052 Gent, Belgium; 3 Department of Information Technology, University of Turku/Turku Centre for Computer Science (TUCS), Turku, Finland; 4 Broad Institute of MIT and Harvard, Cambridge, MA, USA; 5 Department of Mathematical Modelling, Statistics and Bioinformatics, Ghent University, Gent, Belgium

## Abstract

**Background:**

Text mining tools have gained popularity to process the vast amount of available research articles in the biomedical literature. It is crucial that such tools extract information with a sufficient level of detail to be applicable in real life scenarios. Studies of mining non-causal molecular relations attribute to this goal by formally identifying the relations between genes, promoters, complexes and various other molecular entities found in text. More importantly, these studies help to enhance integration of text mining results with database facts.

**Results:**

We describe, compare and evaluate two frameworks developed for the prediction of non-causal or 'entity' relations (REL) between gene symbols and domain terms. For the corresponding REL challenge of the BioNLP Shared Task of 2011, these systems ranked first (57.7% F-score) and second (41.6% F-score). In this paper, we investigate the performance discrepancy of 16 percentage points by benchmarking on a related and more extensive dataset, analysing the contribution of both the term detection and relation extraction modules. We further construct a hybrid system combining the two frameworks and experiment with intersection and union combinations, achieving respectively high-precision and high-recall results. Finally, we highlight extremely high-performance results (F-score *>*90%) obtained for the specific subclass of embedded entity relations that are essential for integrating text mining predictions with database facts.

**Conclusions:**

The results from this study will enable us in the near future to annotate semantic relations between molecular entities in the entire scientific literature available through PubMed. The recent release of the EVEX dataset, containing biomolecular event predictions for millions of PubMed articles, is an interesting and exciting opportunity to overlay these entity relations with event predictions on a literature-wide scale.

## Background

Due to the exponential growth of the biomedical literature, text mining tools have become crucial to process all available information contained in literature databases such as PubMed. Text mining can offer automatically generated summaries to the expert user who needs to retrieve all knowledge on a certain topic or stay up-to-date with recent findings. The level of detail of the extracted information ranges from simple binary interactions, such as protein-protein interactions [1,2] or gene-disease associations [3,4], to a more complex event representation [5-7]. All these relations typically involve one or multiple genes or gene products (GGPs).

GGPs are represented by gene symbols or synonyms and can be linked to database identifiers. For instance, *Esr-1 *refers to Entrez Gene ID 2099. Similarly, the full term *human Esr-1 gene *can be linked to the same ID. However, a complex noun phrase should not always be resolved to the embedded gene symbol. For example, the phrase *Esr-1 inhibitor *refers to an entirely different molecular entity.

Understanding complex noun phrases with embedded gene symbols is thus crucial for a correct interpretation of text mining results [8]. Such non-causal relations between a noun phrase and the embedded gene symbol are being referred to as *entity relations *[9], or, in previous work, *static relations *[10]. This type of relationship may also occur between two different noun phrases within one sentence. Typically, such relations hold between two molecular entities without necessary implication of causality or change. Entity relation types include Equivalence, Locus, Protein-Component, Member-Collection and Subunit-Complex.

The REL supporting task [9,11] of the BioNLP Shared Task (ST) of 2011 [12] was focused on extracting entity relations, contributing to the general goal of the ST to support more fine-grained text predictions. Furthermore, by formally defining these relations, a text mining module is able to establish semantic links between various molecular entities found in text (e.g. inhibitors, promoter constructs, gene families, etc.).

A more detailed explanation of the entity relations and the corresponding datasets is provided in the next section. Additionally, we describe two machine learning frameworks applied to the prediction of such relations. The Turku Event Extraction System (TEES) provides a largely unified extraction approach for all BioNLP ST'11 sub-challenges, with relatively minor adaptations specifically for the REL task. The Ghent Text Mining (GETM) framework on the other hand, contains several novel REL-specific modules, including the deduction and application of semantic similarities between domain terms, measured using latent semantic analysis and a manually annotated corpus.

Further, we show how feature selection techniques, in combination with the GETM framework, can be used to analyse and visualise the most discriminative patterns in the data in a structured fashion, offering valuable insights into the classification challenge. Finally, we analyse the performance and strengths of both frameworks on different datasets, analysing the contribution of both the term detection and the relation extraction modules. We conclude the paper by discussing the usage of entity relations for large-scale integrative data mining tools.

## Data

Entity relations are non-causal relations between a GGP symbol (e.g. *Esr-1*) and a domain term. Domain terms are usually general words denoting biomolecular concepts such as *promoter *or *complex*, occasionally such concepts have a specific name such as *NF-kappaB*. A few examples of entity relations are depicted in Table 1.

**Table 1:** Examples of entity relations

Type of relation	Examples
Equivalence	[human *interleukin 2 *gene] [*TNF-alpha *mRNA transcripts]
Member-Collection	[*Ikaros *family members] [inflammatory cytokine genes] including *TNF, IL-1*, and *IL-6*
Protein-Component	[*alpha globin *regulatory element] [tyrosine] phosphorylation of *STAT1*
Subunit-Complex	[*Myc*-*Max *heterodimer] *p50 *or *relA*, the two major subunits of [NF-kappaB]

Examples of entity relation types, including both embedded and non-embedded cases. GGPs are in italic and domain terms are delimited by square brackets.

There are two related corpora publicly available with annotations for entity relations: the data of the BioNLP ST'11 and the more extensive GENIA relation corpus. The characteristics of these two corpora are summarized in Table 2 and 3. The ST data is divided into three distinct datasets: training (850 abstracts), development (150 abstracts) and test data (260 abstracts) [12]. The training set of the GENIA relation corpus corresponds to the training set of the ST data, and the GENIA relation test data corresponds to the ST development data. In both corpora, valid entity relations involve exactly one GGP and one domain term and both occur within a single sentence. Gold standard relations are provided for the training and development set, allowing the application of machine learning algorithms to produce predictions for the test set.

**Table 2:** Dataset dimensionalities

Relation type	Train instances	Test instances
**Protein-Component (ST)**	1689	334
**Subunit-Complex (ST)**	751	163

**Equivalence (GENIA - E)**	720	129
Functional (GENIA - E)	110	17
Locus (GENIA - E)	11	5
Member-Collection (GENIA - E)	5	0
Misc (GENIA - E)	53	11
Object-Variant (GENIA - E)	14	5
Out-of (GENIA - E)	40	7
**Protein-Component (GENIA - E)**	222	51
**Subunit-Complex (GENIA - E)**	108	22

**Member-Collection (GENIA - NE)**	760	181
**Protein-Component (GENIA - NE)**	593	174
**Subunit-Complex (GENIA - NE)**	275	82

Number of positive instances of the various types in the entity relation corpora. ST refers to the BioNLP'11 Shared Task data, while GENIA refers to the GENIA relation corpus. The latter corpus is further divided into embedded (E) and non-embedded (NE) cases. Datasets sufficiently large for classification analysis are in bold.

**Table 3:** Corpora characteristics

	Relation types	Embedded distinction	Gold GGPs	Gold terms	800 articles	150 articles	260 articles
**Shared Task**	2	no	yes	no	train	dev.	test
**GENIA relation**	4	yes	yes	yes	train	test	-

Characteristics of the two different entity relations corpora. The number of relation types only includes those that were used in the classification experiments.

In the ST data, two types of entity relations are defined. A Subunit-Complex relation holds between a protein complex and its subunits, while a Protein-Component relation is less specific and involves a GGP and its components, such as protein domains or gene promoters. The GENIA relation corpus contains several additional types, including Equivalence and Member-Collection, which expresses a relationship between e.g. a gene family and its members. This corpus is further divided into 'embedded' and 'non-embedded' relations, the first being relations occurring within a noun phrase [13], and the latter containing broader relations between nominals [10]. All these datasets are available at the GENIA project webpage: http://www-tsujii.is.s.u-tokyo.ac.jp/GENIA.

## Methods

A supervised learning framework is a perfect match to deal with entity relations, as statistical properties can be drawn from the grammatical and lexical structures in the training data, providing a way to generate plausible hypotheses on unseen data. In this section we describe two machine learning frameworks developed for the extraction of entity relations: the winning system of the ST 2011, implemented by Turku University [14], and the system that achieved second place, by Ghent University [15].

### TEES

The Turku Event Extraction System (TEES) is a generalized biomedical relation extraction tool based on a unified, extensible graph representation, where word entities constitute the nodes, and edges between them define the relations (or 'events'). The system consists of a pipeline of three main components based on support vector machines (SVMs) [16]. First, entity nodes are predicted for each word token in a sentence. Then, argument edges are predicted for each pair of such nodes. Finally, the resulting graph is 'pulled apart' by classifying subgraphs as actual events/relations or not. These main steps can be followed by several post-processing steps, such as prediction of speculation and negation, or conversion to the ST file format. For a detailed description of the general system, see [17].

#### Parsing

TEES relies heavily on syntactic dependency parses, represented as graphs of word token nodes and dependency edges. The system uses the Charniak-Johnson parser [18] with David Mc-Closky's biomodel [19] trained on the GENIA corpus [20] and unlabeled PubMed articles. The parse trees produced by the Charniak-Johnson parser are further processed with the Stanford conversion tool [21], creating a dependency parse [22]. The parse is the main source of features for all the SVM classification steps.

#### Term detection

The term detection component classifies each word token in the sentence as being a domain term or not. Multi-token terms are always represented by a single token, their syntactic head. For term detection, features are mostly based on dependency paths, generated up to a depth of three and centered on the candidate token. Word tokens have many attributes that are used as features, such as part-of-speech tags, dependency types, the word itself and its stem using the Porter stemmer [23]. All examples are classified with the SVM^*multiclass *^software, using a linear kernel [24].

The term detection module is optimized in isolation, but this optimal F-score may not always be best for overall system performance. A recall boosting step multiplies the negative class weight with a set value, trading precision for increased recall. This results in more entities being available for the edge detection step, and a higher final F-score.

#### Edge detection

After the prediction of domain terms, the edge detection component predicts argument edges between the given GGP names and the predicted terms. One potential edge candidate is generated for each GGP-term pair in a sentence, and these are classified as *Subunit-Complex, Protein-Component, Member-Collection, Equivalence *or negative. For edge detection, features are largely based on the shortest connecting path of dependencies between the two nodes of respectively the GGP and the domain term. All examples are classified with the same SVM software as for term detection. Since entity relations are pairwise, no further processing is required, and the resulting graph can be directly converted to the ST format.

### GETM

**Figure 1:**
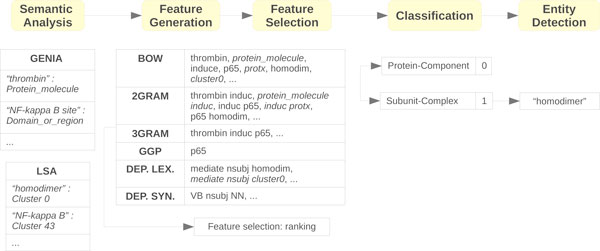
**Flowchart of the GETM framework**. Flowchart of the GETM framework, including example intermediate steps for the sentence "Thrombin-induced *p65 *homodimer binding to downstream NF-kappa B site of the promoter mediates endothelial ICAM-1 expression and neutrophil adhesion."

The GETM framework is based on a previously introduced event extraction system [7] which was significantly extended with REL-specific modules. It first calculates semantic similarities between domain terms. These similarities are used to construct generalized feature vectors that represent the semantic and grammatical information contained in the training sentences. The rich feature vectors are then subjected to feature selection and subsequently used for training a binary SVM for each entity relation type. Finally, for each selected sentence and each GGP occurrence, a suitable domain term is selected within a certain search window. The flowchart of the GETM framework is depicted in Figure 1.

In contrast to TEES, the GETM framework, with its REL-specific modules, has not been published yet (with the exception of a short poster abstract [15]). In the next sections, we therefor describe the GETM REL-specific modules in a bit more detail.

#### Semantic analysis

To fully understand the relationship between a GGP and a domain term, it is necessary to account for the usage of synonyms and lexical variants in human language. To capture this textual variation, two strategies were implemented for creating semantic lexicons, grouping similar words together. The first method takes advantage of manual annotations of semantic categories in 1000 articles. The second method relies on statistical properties of nearly 15,000 articles.

The GENIA term corpus contains manual annotations of various domain terms such as promoters, complexes and other biological entities [25]. These annotations are used to link certain lexical patterns to semantic categories, such as *DNA-domain-or-region *and *protein-family-or-group*. The GENIA term corpus consists of the same 1000 abstracts as the combined training and development ST data, and is therefore a very suitable additional data source.

In addition to using the GENIA term corpus, semantic spaces were calculated to deduce the underlying similarities between domain terms. A semantic space can be defined as a mathematical representation of a text corpus, containing high-dimensional vectors that capture the context in which certain words are used. Similar vectors then represent semantically similar words. By applying semantic spaces in this study, we aim at finding clusters of closely related biomolecular concepts, such as *complex *and *heterodimer*.

In a first step, a large-scale corpus is collected containing 14,958 PubMed articles concerning the topic of the GENIA corpus: human transcription factor blood cells. Next, all words are transformed to their lowercase variants and the Porter stemming algorithm is used for generalization purposes [23].

The actual semantic spaces are then built with the open-source S-Space Package [26]. This package contains implementations of several semantic algorithms that have been extensively documented, tested and validated. We have experimented with latent semantic analysis (LSA) [27], random indexing [28], HAL [29] and COALS [30]. By running these semantic algorithms on the nearly 15 thousand articles, we obtain datasets of terms linked to their semantic vectors. In a final step, these semantic vectors are clustered into meaningful groups. Clustering was performed using the Markov Cluster algorithm [31] with the cosine similarity measure.

To assess the best fitting semantic algorithm for this specific classification task, a score heuristic *S *was implemented to evaluate the resulting clusters:

(1) S=Unknown×HG×Reliability

(2) $$Reliability=\frac{Known}{Known+Unknown}$$

Some terms in the clusters can be assigned a gold classification label by looking at the training portion of the GENIA relation corpus. For example, the domain term "complex" is always associated with a Subunit-Complex relation. The number of such gold labels in each cluster is represented by *Known *and the homogeneity of a cluster (*HG*) expresses the internal agreement between these labels. The homogeneity *HG *is multiplied by the number of unlabeled test terms (*Unknown*) to assess the predictive value of the cluster. The reliability (*Reliability*) of the cluster further expresses the percentage of known labels versus predicted ones. Clusters with relatively more known labels are deemed to be more reliable, unless the labels are highly contradicting, which would result in less homogeneity and thus a lower score. The final score metric *S *calculates one score for each cluster, and a clustering result is scored as the sum of all clusters.

**Figure 2:**
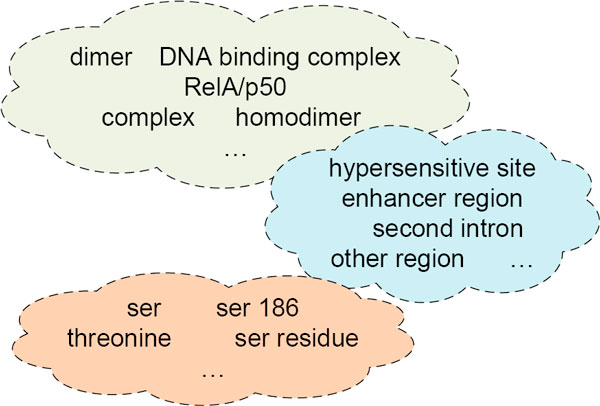
**Clustering example**. A few examples of clusters of domain terms, derived by LSA and clustered with the Markov Cluster algorithm.

Evaluation using the score heuristic *S *clearly indicates that the semantic algorithms random indexing and HAL produce less useful results than LSA and COALS. After manual inspection of the clusters, LSA was chosen as the preferred method to produce semantic vectors. Figure 2 depicts some of the resulting clusters.

#### Machine learning module

The machine learning component of the framework identifies entity relations by analysing lexical and grammatical patterns in sentences containing GGPs.

To capture the lexical information for each sentence containing at least one GGP, bag-of-word (BOW) features are derived. In addition, n-grams, containing *n *consecutive words, are extracted from the sentence. All lexical information in the feature vectors is stemmed with the Porter algorithm and generalized by blinding the GGP symbol with *protx *and all other co-occurring GGP symbols with *exprotx*, while at the same time keeping the content of these symbols as separate features. Furthermore, terms occurring in the semantic lexicons (as described previously) are blinded with the corresponding cluster number or category for additional, generalized features.

For extracting grammatical patterns, the same parsing techniques are employed as used by TEES. Additionally, the semantic mappings are applied to the patterns derived from dependency graphs, and one additional generalization is obtained by substituting words for their part-of-speech tags. A few example features are depicted in Figure 1, with generalized features in italic.

**Figure 3:**
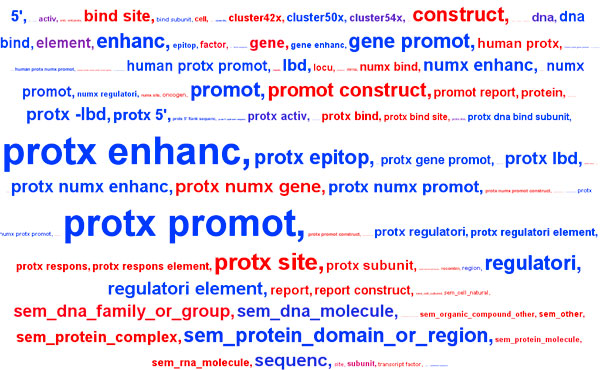
**Feature selection analysis for Protein-Component**. Most important features for predicting Protein-Component relations, as predicted by the GETM framework. The feature cloud shows all types of grammatical and lexical features that are most discriminative according to the ensemble feature selection algorithm. Red indicates features that mark negative examples, blue features mark positive examples.

To reduce the dimensionality of the resulting datasets, ensemble feature selection is performed with linear SVMs as described in [32]. This feature selection methodology has been shown to enhance performance of text mining tools in a supervised learning setting and can also serve to gain a better insight into the task at hand. Hence, feature clouds have been automatically generated and manually analysed to improve the feature generation module. For example, Figure 3 depicts the feature cloud of the most informative feature patterns when predicting embedded Protein-Component relations for the GENIA relation dataset. Features indicating positive examples (blue) include words of the semantic class 'protein-domain-or-region' and the lexical pattern (trigram) 'human *protx *promoter'. Negative features (bright red) include the 2-gram '*protx *subunit' and the semantic class 'protein-complex', which would in turn be a positive hint for the Subunit-Complex type. Notably, there are almost no syntactic features in the top most informative features. This is a property inherent to the prediction of the embedded class of entity relations, for which the close lexical context of the GGP is the most determining factor. In contrast, the non-embedded types do rely more on the syntactic structure of the sentence.

The final feature vectors are classified using an SVM with a radial basis function (RBF) as kernel. The RBF kernel has been evaluated to perform best in this framework which employs several binary, type-specific classifiers in parallel [7]. An optimal parameter setting (*C *and *gamma*) for this kernel was obtained by 5-fold cross-validation on the training data.

#### Term detection

In the GETM framework, sentences are selected for classification if they contain at least one GGP. When the sentence is classified as containing a certain type of entity relation, it is necessary to also identify the exact domain term that is related to the GGP. To this end, a pattern matching algorithm was designed that applies a rule-based search algorithm within a given window (number of tokens) around the gene symbol. The search algorithm employs dictionaries obtained from the training data in combination with information from the semantic lexicons.

## Results and discussion

In this section we first present the official ST'11 results. We then analyse these results and the underlying frameworks in more detail by benchmarking on the GENIA relation corpus. A hybrid framework is created and validated on the (hidden) ST test set. Finally, we experiment with further combinations of the frameworks and achieve either high-precision or high-recall results.

### Official results of the ST'11

Table 4 depicts the performance of the official submissions for the REL subtask of the BioNLP Shared Task of 2011. TEES obtained a first place with an F-score of 57.71% [14]. The GETM framework achieved a global performance of 41.62% F-score [15], ranking second. Concordia University ranked third with 32.04% F-score [33].

**Table 4:** Results on the ST data

	Subunit-Complex			Protein-Component				All	
	**Prec**.	**Rec**.	**F**	**Prec**.	**Rec**.	**F**	**Prec**.	**Rec**.	**F**

**TEES**	66.95	48.47	56.23	68.57	50.90	58.43	68.04	50.10	**57.71**
**GETM**	38.12	47.85	42.43	36.53	47.31	41.23	37.04	47.48	**41.62**

**Hybrid**	66.95	48.47	56.23	61.79	52.40	56.70	63.32	51.11	**56.56**

**T ∩ H**	75.25	46.63	57.58	71.56	48.80	58.03	72.70	48.09	**57.89**
**T ∪ H**	60.74	50.31	55.03	59.73	53.89	56.66	60.05	52.72	**56.14**

Performance on the ST'11 test set, measured by precision, recall and their harmonic mean, the F-score (F). The first few rows indicate the official results of the TEES (T) and GETM frameworks. Next, the performance of the hybrid (H) system is shown. Finally, the two last rows report on the performance of creating the intersection and the union of TEES and the hybrid system.

A performance gap of 16 percentage points is measured between the best and second system, and another discrepancy of 9.5 percentage points between the second and third system. The relatively high performance of TEES is remarkable, as this system has not been developed specifically for the detection of entity relations, but rather is able to generalize quite well to different text mining challenges. In contrast, the GETM framework contains specific algorithms designed for the entity relations classification task such as the creation of the semantic lexicons. In this study, we aim at elucidating the performance discrepancy between the first two systems, by analysing whether most errors originate from the term recognition step or from the relation extraction module.

### Analysis on the GENIA relation corpus

To analyse the performance discrepancy between TEES and the GETM framework, a number of analyses were performed on the GENIA relation corpus. The GENIA relation corpus was chosen for two main reasons. First, its scope is broader and the annotations cover several additional types of entity relations compared to the ST data (Table 2). Secondly, the availability of gold standard domain annotations in the GENIA relation corpus allow for benchmarking the relation extraction module in isolation. This also means that the results obtained here are not directly comparable to the results on the ST data, because the latter corpus does not include gold domain terms.

For the new experiments, TEES has remained unchanged, while the feature generation module of the GETM framework has been modified slightly to benefit from the specific properties of the GENIA relation corpus, optimizing feature sets for the different entity relation types (embedded vs. non-embedded). Due to the available gold standard terms, the GETM framework now classifies sentences with exactly one GGP and one term, rather than just sentences containing one GGP. Consequently, additional features describing the lexical and semantic content of the domain terms are added to the feature vectors.

The classification parameters of TEES have been optimized on the ST development corpus, which corresponds to the GENIA relation test set. For the GETM framework, the best feature set was selected after several analyses on the same data. These settings result in slightly optimistic performance values for both systems, when benchmarking on the GENIA relation data. However, the resulting overfitting only accounts for a few percentage points in F-score, and because these analyses are used for comparison between TEES and GETM, this is not considered to be a problem. This is even more the case because the hidden ST test set is the *de facto *standard for benchmarking and comparing different systems. The results on this dataset are described in the next section.

For the classification experiments on the GENIA relation corpus, separate runs were performed for 'embedded' and 'non-embedded' relations. The performance results are depicted in Table 5. From this table, we learn that both frameworks perform almost equally well, with a small advantage for TEES. The huge discrepancy, as observed in the official results, has disappeared. This can be explained by the availability of the gold standard domain terms, but may also be due to the added relation types. For example, GETM performs worse than TEES for the Subunit-Complex relation type in both the ST and GENIA evaluations, but performs better for the Equivalence type, which is not included in the ST evaluation. To further isolate and analyse the influence of the term detection module specifically, we will create a hybrid framework and evaluate it on the ST corpus in the next section.

**Table 5:** Results on the GENIA relation corpus

	TEES			GETM		
**Relation type**	**Prec**.	**Rec**.	**F**	**Prec**.	**Rec**.	**F**

**Equivalence (E)**	93.13	95.31	94.21	97.64	96.12	96.88
**Protein-Component (E)**	96.08	96.08	96.08	100.00	86.27	92.63
**Subunit-Complex (E)**	79.17	86.36	82.61	80.00	72.73	76.19
**All (E)**	92.23	94.53	**93.37**	97.85	90.10	**93.81**

**Member-Collection (NE)**	81.44	75.14	78.16	71.73	75.69	73.66
**Protein-Component (NE)**	87.77	67.03	76.01	73.33	83.63	78.14
**Subunit-Complex (NE)**	81.54	64.63	72.11	73.24	63.41	67.97
**All (NE)**	83.83	69.89	**76.23**	72.65	76.50	**74.52**

**ALL (E+NE)**	86.83	77.55	**81.93**	79.94	80.82	**80.38**

Performance on the GENIA relation corpus for embedded (E) and non-embedded (NE) relation types, measured by precision, recall and their harmonic mean, the F-score.

Another important result emerging from the analysis on the GENIA relation corpus, is the performance discrepancy between embedded and non-embedded types. Global performance reaches around 93-94% F-score for the embedded cases, while the non-embedded relations are predicted with an F-score of 74-76%. The embedded cases are indeed less grammatically complex than the non-embedded ones. Interestingly, they do represent an important sub-challenge of entity relations. When combining text mining results with public databases, automatically tagged GGP symbols need to be resolved to the correct record in the database. GGP symbols are often extracted by named entity recognition software such as BANNER [34], which applies statistical models for the recognition of GGP symbols in text, and might sometimes tag a whole noun phrase rather than just the embedded GGP name. Embedded relation types formally describe the relationship between e.g. *Esr-1 *and *Esr-1 promoter*, thus providing an automatic way of dealing with these strings and enabling a meaningful integration between text and database records. Even taking the previously described effects of overfitting into account, embedded relations can still be predicted with an F-score above 90%.

The recent release of the EVEX dataset, containing biomolecular event predictions for millions of PubMed articles [35,36], provides an interesting opportunity to overlay these entity relations with event predictions. In this setting, entity relations could provide hubs between events concerning similar molecular entities, they could improve on the level of detail provided by the events [8] and finally they would be useful for large-scale normalization and integration with external databases such as Entrez Gene from NCBI [37] or Uniprot [38].

### Combining the two frameworks

To test the hypothesis that the GETM framework lags behind because of its term detection module, a hybrid framework was created by combining the term detection module of TEES with the GETM relation detection module. This framework is tested on the official ST test data and it performs almost equally well as the original submission by TEES (1.15 percentage points lower F-score, Table 4). This result clearly shows the huge impact of the term detection module on the final results, as the relation extraction modules perform almost equally well. Apparently, the SVM-based term detection module of TEES performs much better than the rule-based approach implemented in the GETM framework, resulting in a much higher global performance result on the ST data.

Even though the performance of TEES and the hybrid framework are very similar, there is still a considerable variability in the underlying predictions, as the relation extraction component differs significantly. Consequently, we can further experiment with ensemble methods to combine both systems. Considering we only have access to two high-performing systems, the options for creating combinations are limited.

First, the intersection of the two systems was created. Comparing two relations across the different frameworks is straightforward because they use the same GGP occurrences (gold annotations) and the same domain terms (predicted by TEES). The results are shown in Table 4. Obviously, an intersection could never improve on recall compared to the original submission by TEES, but we do find a precision increase of 2.99 and 8.30 percentage points for Protein-Component and Subunit-Component respectively. The resulting F-score is 0.19 percentage points higher. While this is marginally higher than the original submission with TEES, the difference is not statistically significant, and this new framework is more complex as it needs to train two different classifiers. Finally, it is important to note that any machine learning framework can in theory be tuned to achieve either high recall or high precision by applying the well-known precision-recall trade-off [39].

The union of TEES and the hybrid system was subsequently constructed aiming at higher recall rates while still benefiting from the relatively high precision rates of both systems. However, this approach seems to include many irrelevant false positives (Table 4, last row). Recall rises with 2.99 and 1.84 percentage points for Protein-Component and Subunit-Component respectively, but F-score drops with 1.57 percentage points compared to the original submission with TEES.

## Conclusions

We have presented the two best frameworks for the BioNLP ST'11 REL challenge, discussing the application of machine learning techniques, semantic spaces and feature selection for this task. We further analysed the performance discrepancy of 16 percentage points between these two frameworks as observed on the ST data. Benchmarking on a related and more extensive dataset has guided the construction of a hybrid framework which combines the TEES term recognition module with the GETM relation detection module. From these experiments, it became clear that the term detection module has a much higher impact than the relation extraction module on the final performance, and future development efforts in this field should thus focus more on accurate detection of the domain terms.

The extraction of entity relations from text has several interesting applications, such as retrieval and semantic labeling of various molecular concepts within and across articles. Additionally, the prediction of entity relations can also have an impact on the prediction of biomolecular events from text. Finally, knowledge on entity relations is a crucial step for the normalization of biological entities automatically extracted from text with named entity recognition software. We have shown that we can predict the class of embedded entity relations, necessary for such normalization efforts, extremely well (F-score above 90%).

The results obtained in this study will enable us in the near future to annotate semantic relations between molecular entities in the entire scientific literature available through PubMed, exploiting these relations for further refinements and improvements of large-scale text mining efforts.

## Competing interests

The authors declare that they have no competing interests.

## Authors' contributions

JB and SVL have designed and implemented the TEES and GETM frameworks respectively. TA has contributed to GETM with algorithms and analysis for clustering and feature selection. SVL, TA and JB have drafted the manuscript. BDB, TS and YVdP have participated in the design and coordination of the study. All authors have read and approved the final manuscript.
